# Insertion Sequence–Driven Diversification Creates a Globally Dispersed Emerging Multiresistant Subspecies of E. faecium

**DOI:** 10.1371/journal.ppat.0030007

**Published:** 2007-01-26

**Authors:** Helen L Leavis, Rob J. L Willems, Willem J. B van Wamel, Frank H Schuren, Martien P. M Caspers, Marc J. M Bonten

**Affiliations:** 1 Eijkman–Winkler Institute for Medical Microbiology, Infectious Diseases and Inflammation, University Medical Center Utrecht, Utrecht, The Netherlands; 2 TNO Quality of Life, Department of Microbiology, Zeist, The Netherlands; Harvard Medical School, United States of America

## Abstract

*Enterococcus faecium,* an ubiquous colonizer of humans and animals, has evolved in the last 15 years from an avirulent commensal to the third most frequently isolated nosocomial pathogen among intensive care unit patients in the United States. E. faecium combines multidrug resistance with the potential of horizontal resistance gene transfer to even more pathogenic bacteria. Little is known about the evolution and virulence of *E. faecium,* and genomic studies are hampered by the absence of a completely annotated genome sequence. To further unravel its evolution, we used a mixed whole-genome microarray and hybridized 97 E. faecium isolates from different backgrounds (hospital outbreaks (*n* = 18), documented infections (*n* = 34) and asymptomatic carriage of hospitalized patients (*n* = 15), and healthy persons (*n* = 15) and animals (*n* = 21)). Supported by Bayesian posterior probabilities (PP = 1.0), a specific clade containing all outbreak-associated strains and 63% of clinical isolates was identified. Sequencing of 146 of 437 clade-specific inserts revealed mobile elements (*n* = 74), including insertion sequence (IS) elements (*n* = 42), phage genes (*n* = 6) and plasmid sequences (*n* = 26), hypothetical (*n* = 58) and membrane proteins (*n* = 10), and antibiotic resistance (*n* = 9) and regulatory genes (*n* = 11), mainly located on two contigs of the unfinished E. faecium DO genome. Split decomposition analysis, varying guanine cytosine content, and aberrant codon adaptation indices all supported acquisition of these genes through horizontal gene transfer with IS*16* as the predicted most prominent insert (98% sensitive, 100% specific). These findings suggest that acquisition of IS elements has facilitated niche adaptation of a distinct E. faecium subpopulation by increasing its genome plasticity. Increased genome plasticity was supported by higher diversity indices (ratio of average genetic similarities of pulsed-field gel electrophoresis and multi locus sequence typing) for clade-specific isolates. Interestingly, the previously described multi locus sequence typing–based clonal complex 17 largely overlapped with this clade. The present data imply that the global emergence of *E. faecium,* as observed since 1990, represents the evolution of a subspecies with a presumably better adaptation than other E. faecium isolates to the constraints of a hospital environment.

## Author Summary

Whole-genome sequencing has become instrumental in investigating the genome contents of bacteria. However, there is enormous diversity within bacterial populations, and annotation of multiple genomes is costly and elaborate. For investigating diversity and phylogeny within bacterial species, comparative genomic hybridization is an attractive alternative that may provide fundamental insights into the factors (genes) distinguishing bacterial subpopulations. *Enterococcus faecium,* a worldwide emerging nosocomial pathogen usually resistant to multiple antibiotics, causes infections in immunocompromised patients. Using comparative genomic hybridization of 97 E. faecium strains isolated from different epidemiological niches worldwide, a subpopulation of E. faecium strains was identified that was associated with invasive infections and hospital outbreaks. Approximately 13% of the E. faecium pangenome was highly specific for this subpopulation, and, based on phylogenetic clustering, it should be considered a subspecies. We hypothesize that extensive variation within specific functional genes and high prevalence of mobile elements, mostly insertion sequence elements, contributed to the success of this genetic subset in its competition with other enterococci in hospital settings, creating a novel globally dispersed nosocomial subspecies. These findings fully confirmed previous phylogenetic studies based on multi locus sequence typing that had also revealed a genetic subset of *E. faecium,* clonal complex 17. Identification of genes specific for clonal complex 17 is a first step in elucidating how global spread and adaptation to the hospital environment of this emerging nosocomial pathogen has occurred.

## Introduction

Once not recognized as clinically relevant microorganisms, enterococci currently are the third most frequently isolated nosocomial pathogen from intensive care unit patients in the United States [1]. The emergence of enterococci as nosocomial pathogens in the 1990s was associated with a gradual replacement of Enterococcus faecalis by Enterococcus faecium and an epidemic rise of vancomycin-resistant E. faecium [2]. In Europe, though, vancomycin-resistant enterococcus (VRE) initially was only found to colonize healthy individuals, and nosocomial VRE outbreaks have only recently begun to emerge. This epidemiological difference between the US and Europe presumably resulted from massive bioindustrial avoparcin usage in Europe, which created a VRE reservoir among farm animals with spillover via the food chain to consumers [3–8]. Abundant antibiotic use in hospitals, most notably of vancomcyin and cephalosporins, was the presumed cause of VRE emergence in US hospitals [9].

The emergence of VRE as a nosocomial pathogen in countries with polyclonal endemicity seems irreversible, despite enforced hygiene measures and restricted antibiotic prescription policies [10]. Nevertheless, despite unsuccessful eradication, a sustained reduction in prevalence rates has been reported [11]. Also, successful control of monoclonal outbreaks in countries with low VRE prevalence has been reported [12]. Recent reports on the transfer of vancomycin resistance from enterococci to methicillin-resistant Staphylococcus aureus [13–16] stressed the need to better understand molecular epidemiology, as well as transmissibility and virulence of enterococci, to control further spread and develop treatment and eradication strategies. Yet, little is known about the virulence and pathogenesis of E. faecium. Apart from antibiotic resistance genes, only the *enterococcal surface protein (esp)* gene and the *hyaluronidase* gene have been epidemiologically associated with infections and documented hospital outbreaks [17–20]. The *esp* gene is contained on a putative pathogenicity island (PAI), but functional studies on any of these E. faecium genes have not been performed yet [18]. Previously, we described the population structure of E. faecium with multi locus sequence typing (MLST), relying on the variation in silent mutations in short sequences from seven housekeeping genes [21,22]. This population shows host specificity, and a globally present hospital subpopulation, clonal complex (CC) 17, is responsible for most outbreaks and colonization of hospitalized patients [22]. Apart from linkage with the putative PAI, not much is known about the gene content of this subset and whether, based on gene content, a similar population can be characterized.

Microarray-based comparative genomic hybridization (CGH) has provided novel insights into the diversity and adaptability of several bacterial populations, such as the relevance of lateral gene transfer and recombination, which both result in mosaic genome structures in Helicobacter pylori [23,24], *Salmonella* species [25], Escherichia coli [26,27] and S. aureus [28–30]. In addition, CGH has been used to study evolution and to decipher bacterial virulence and host specificity [31–33]. In this respect, CGH has major advances over more conventional genotyping methods, as it also provides insights into the core genome and accessory genes, which may help to further disclose gross signatures of niche differentiation. Almost all CGH studies originate from PCR-based arrays of amplified open reading frames (ORFs) derived from one or multiple annotated sequenced strains, sometimes completed with additional genes not present in the sequenced strains. This approach, though expensive, ascertains coverage of a whole genome. Unfortunately, this approach is not possible for *E. faecium,* as there is no complete annotated genome sequence. Moreover, the partially sequenced, but still not annotated, E. faecium DO strain doesn't contain the putative PAI, one of the few known gene clusters associated with virulence and epidemicity. For all these reasons, a different approach is necessary for a broad and detailed genomic analysis of E. faecium.

In the present study, we performed comparative phylogenomics to study the genome composition population-wide as well as population dynamics of E. faecium using a mixed whole-genome array constructed from a shotgun library of nine strains from different ecological and genetic backgrounds, including the sequenced E. faecium DO strain. DNA–DNA hybridizations of 97 epidemiologically and genotypically different isolates to the array identified a distinct, globally dispersed clade containing all epidemic isolates and the majority of clinical isolates. Isolates within this clade harbored a large content of accessory genes mainly concentrated on two contigs in E. faecium DO. Furthermore, hybridization data revealed high rates of recombination and deletion resulting in mosaic-structured genomic regions. Insertion sequence (IS) elements were predicted to be prominent loci in the bifurcation of this clade, and probably have played a major role in adaptation and diversification of hospital-associated E. faecium strains belonging to this clade.

## Results

### Array Evaluation

In total, 3,474 spots (*n*(genomic[g]) = 2,727 , *n*(plasmid[p]) = 712, *n*(extra spotted genes) = 35) met the quality criteria (see Materials and Methods) and were included in this study. Since the microarray consists of a mixture of nine strains, only the genomic coverage of core genes can be determined. The total nucleotide and gene detection coverage of the E. faecium core genome was estimated to be 80% and 93%, respectively, using algorithms by Akopyants et al. and Moore et al., respectively [34,35]. Obviously, the microarray coverage of strain-unique accessory genes will be lower.

The detected Cy5/Cy3 ratios of the 3,474 spots were subjected to log2 transformation and GACK normalization (see Materials and Methods). Duplo hybridizations of seven isolates clustered as nearest neighbors in hierarchical clustering, each with 92% identical GACK values (98% for binary output) in spot profile, indicating that microarray results were highly reproducible. Log2-transformed data is available in Dataset S1. Validation of six ORFs located on ten inserts by Southern hybridization followed the presence and absence of spots-transformed array data (unpublished data; see Materials and Methods).

Among a selection of the accessory genome of 437 clone inserts, a subselection of 146 inserts (explained elsewhere in this section) was partially sequenced for further analysis. Sequence alignments revealed 16 redundant genes from 42 inserts and seven redundant plasmid loci from 27 inserts (represented by >100 base pair–overlap in two to four spots). Eight of these 16 genes (27 of 42 inserts) were present in multiple copies in the E. faecium DO genome; therefore, actual redundancy among the libraries is limited.

### Comparative Phylogenomic Analysis Based on Origin

**Figure 1:**
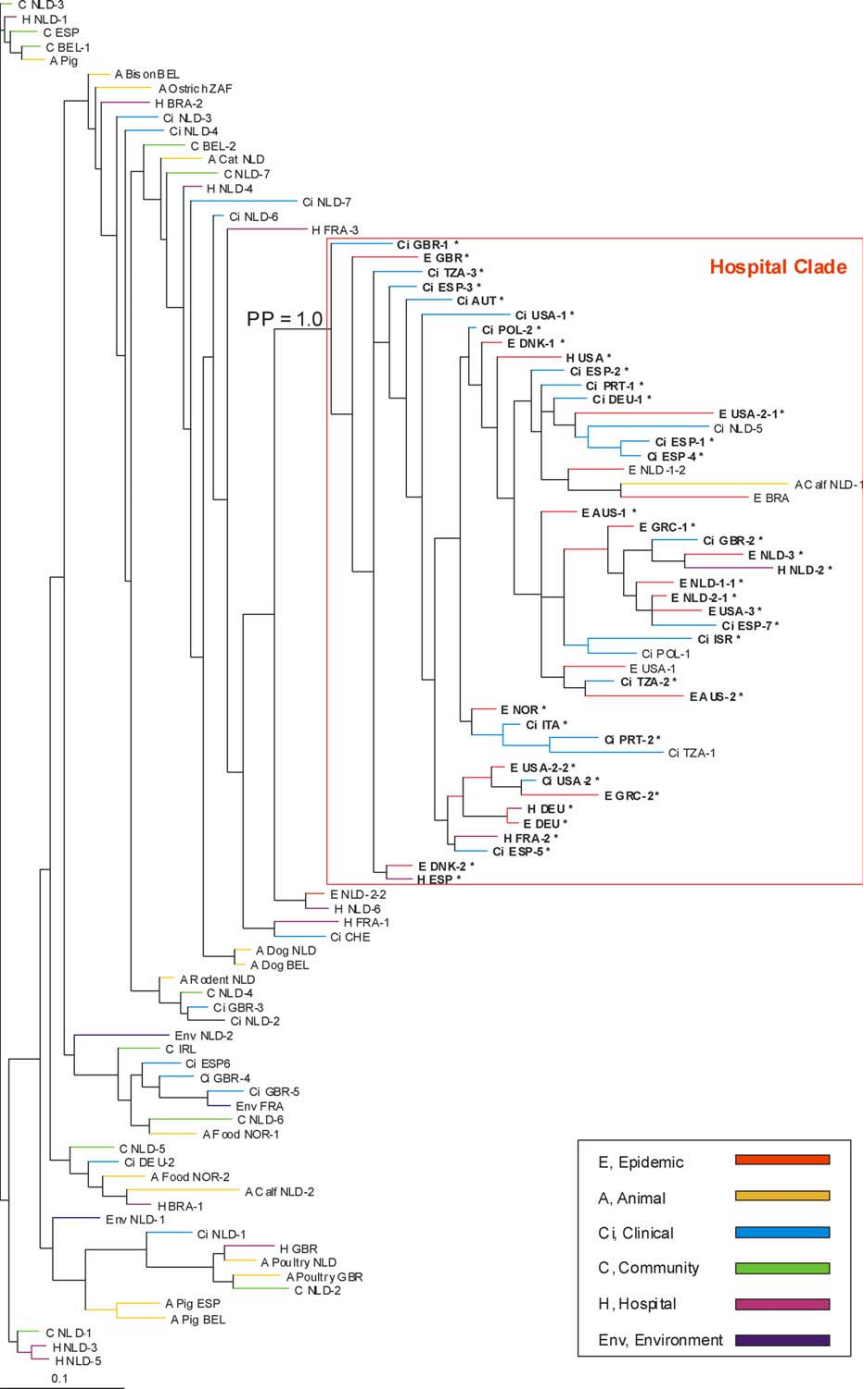
A Bayesian Phylogenomic Relationship of Strains Associated with Different Ecological Niches The Bayesian posterior probability (PP) supports internal branch robustness. PP = 1.0 represents 100% of all phylogenies showing a given topology. Strains are designated at the end of branches and are colored according to the ecological niche from which the E. faecium strain was isolated. Strains in bold indicated with an asterisk are part of CC17. AUS, Australia; AUT, Austria; BEL, Belgium; BRA, Brazil; CHE, Switzerland; DEU, Germany; DNK, Denmark; ESP, Spain; FRA, France; GBR, Great Britain; GRC, Greece; IRL, Ireland; ISR, Israel; ITA, Italy; NLD, Netherlands; NOR, Norway; POL, Poland; PRT, Portugal; TZA, Tanzania; USA, United States of America; ZAF, Republic of South Africa.

Phylogenomic analysis with the microarray data using a Bayesian-based phylogenic method identified a distinct clade (Bayesian posterior probabilities [PP] = 1.0) containing all epidemic isolates (*n* = 18), 63% of clinical isolates (*n* = 22/35), 33% of hospital surveillance isolates (*n* = 5/15), no community survey isolates, 7% (*n* = 1/15) of animal isolates, and 0% (*n* = 0/3) of environmental isolates (Figure 1 and Table 1). E. faecium DO (E1794 in Table 1) is also contained in this clade. Throughout the rest of the article this clade is referred to as *hospital clade,* and hospital-associated E. faecium strains belonging to this clade are referred to as *hospital clade strains*. The bifurcation was supported by complete linkage transversal hierarchical clustering with GACK-transformed data of graded output, and by maximum parsimony analysis on binary GACK-transformed data (Table 1). With the latter two techniques, only one isolate was clustered differently. Internal branching within and outside this specific clade was less reliable (mostly PP = 0.50). The identification of a distinct hospital clade indicates the successful evolution of an E. faecium clone that adapted to its niche and diversified.

**Table 1:**
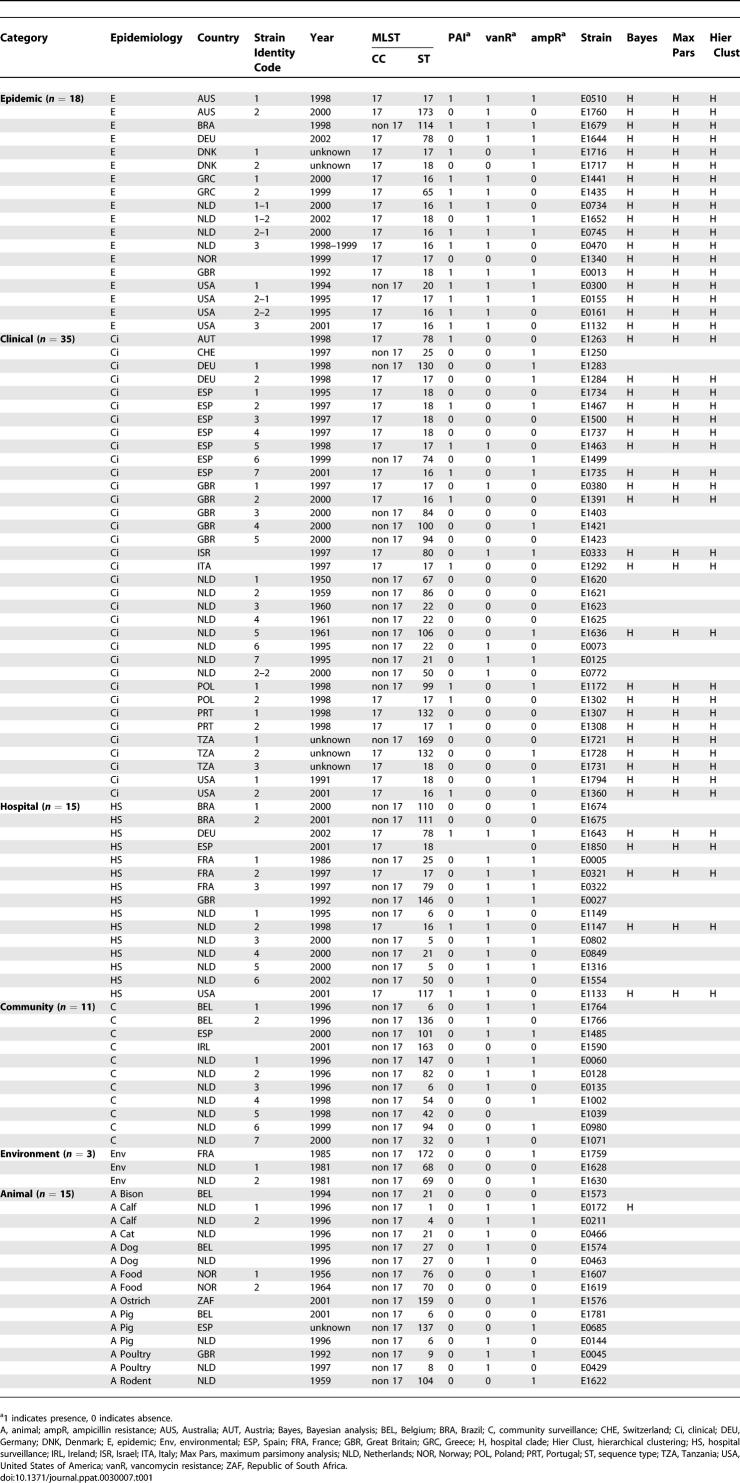
Strains Used for Hybridization

### Gene Composition of the E. faecium Pangenome and Identification of Genes Specific to the E. faecium Hospital Clade

**Figure 2:**
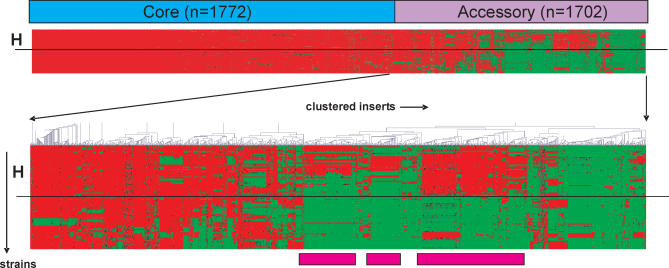
Complete Linkage Hierarchical Clustering of E. faecium Inserts with Pearson Correlation and Euclidian Distance, Zoom on Accessory Insert The hierarchical clustering of inserts visualizes composition of the microbial pangenome: the core genome and accessory genes. The lower panel of the figure represents a zoomed-in accessory genome, as indicated by the vertical and diagonal arrows in the middle of the figure. Presence of an insert is indicated in red and absence of an insert in green. Inserts are clustered on the *x*-axis, and strains are presented on the *y*-axis (indicated by a vertical arrow left of the lower panel). Horizontal straight lines separate the hospital clade strains (H) from the non-hospital clade strains. The core genome and accessory genes are indicated by a blue and purple bar, respectively. Hospital clade–associated clustered inserts are indicated with pink bars.

The E. faecium core genome defined as genomic spots present or divergent (GACK > −0.50) in each of the strains consisted of 65% of all genomic spots (*n*(g) = 1,772) (Figure 2). The clone inserts of 35 randomly selected spots were PCR-amplified, partially sequenced, and blasted to GenBank; these inserts encoded 37 (partial) genes (Table S1). Twenty-seven inserts showed highest similarity with 30 different E. faecium DO genes located on different contigs, two with two different E. faecium DO sequences with no corresponding ORF, and six with 12 *E. faecalis* V583 genes (Table S1). Because of array design (random shearing), more than one (partial) gene could be located on one insert. Assigned functions by clusters of orthologous genes (COGs) defined genes to be involved in basic cell function (Table S1).

Among all other spots (*n*(g) = 955, *n*(p) = 712, *n*(extra spotted genes) = 35) representing the accessory genome, 437 spots (*n*(g) = 165, *n*(p) = 261, *n*(extra spotted genes) = 11) were ≥80% specific for and significantly associated with the hospital clade (χ^2^ test followed by false discovery rate [FDR] correction (*p* < 0.01)) (Figure 2). The sensitivities for presence of these spots in clade-specific strains varied from 20% to 98%, indicating that some spots were present in almost all isolates belonging to the hospital clade, while other inserts were only present in a small subset (Table 2). The inserts from a selection of 146 spots (*n*(g) = 86, *n*(p) = 60) (criteria in Materials and Methods) were partially sequenced and blasted in GenBank for significant similarity. Sequencing revealed 175 ORFs with varying similarity to genes present in the E. faecium DO genome (131 ORFs located on 104 inserts), on E. faecium plasmids (21 ORFs on 19 inserts), in E. faecalis V583 (six ORFs on eight inserts), and in other bacterial species (17 ORFs) (see Table 2). Nine sequences showed no significant similarity at all (Table 2). Furthermore, 11 separately spotted PCR products were identified as hospital clade–specific. Hospital clade–specific sequences were identical or similar to genes from 13 different COGs (Table 2). By far the largest COG, group L consists of 80 ORFs (46% of 175 ORFs) and contains genes involved in DNA replication, recombination, and repair. This group mainly comprises IS elements and transposases (*n* = 42) and plasmid DNA sequences (*n* = 26). COG groups R and S, representing genes with a general function prediction and unknown function, respectively, are the second most prominent COG groups and include 55 (31%) of the ORFs. ORFs identical or similar to genes encoding metabolic pathways (COG G, E, F, H, P), and to proteins involved in cell wall and membrane biogenesis (M) and transcription (K), occurred less frequently (17, 9, and 12 times, respectively). Among all hospital clade–enriched ORFs, eight inserts represented five different antibiotic resistance genes (streptomycine adenylyltransferase; aminoglycoside phosphotransferase, which is similar to aph(3′)-III; chloramphenicol O-acetyltransferase; an aminoglycoside-streptothricin resistance cassette [*aadE* and *sat4* from the *aadE-sat4-aphA* cluster], and an aminoglycoside resistance cassette [*aac(6')-Ie-aph(2")-Ia* and *aac(6')-Ie-aph(2")-Ia2*]). Eleven ORFs were identical or highly similar to six different (putative) phage genes. Thirty-four of the 261 ORFs that originated from the plasmid library (58%) were similar to gene sequences on two enterococcal plasmids, pEFNP1 and pKQ10.

**Table 2:**
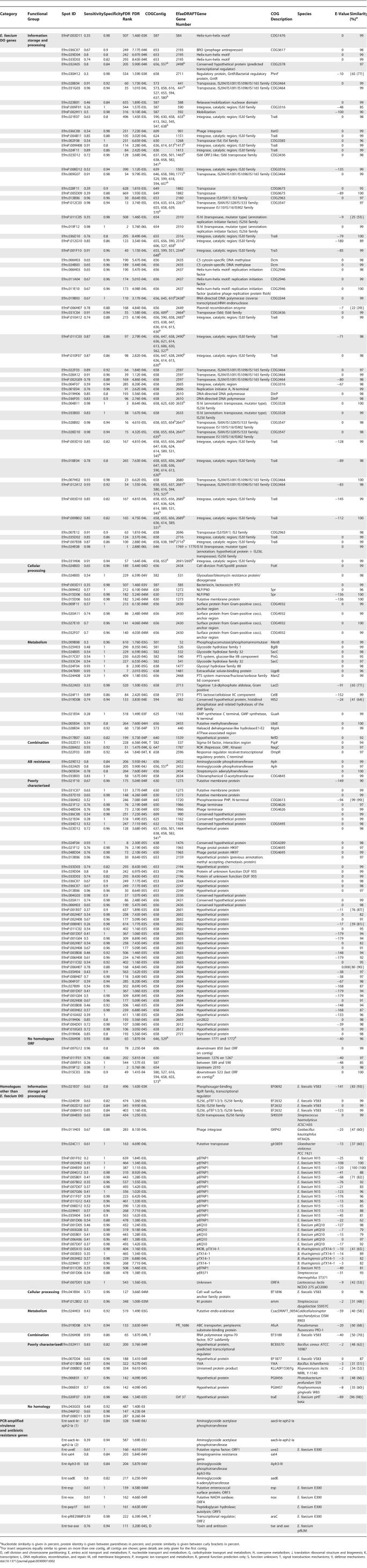
Sequenced E. faecium Hospital Clade–Specific Genes Sorted by Functional COG Groups

At least three different hybridization patterns could be recognized among hierarchical clustering of these plasmid-specific inserts, indicating existence of at least three different pKQ10/pEFNP1 variants.

### Recombination and Genomic Mosaicism

Four different approaches were used to identify recombination in the hospital clade strains. First, we studied patterns of presence and absence of hospital clade–specific genes within gene clusters on the bacterial chromosome. Second, reticular networks were identified with split decomposition analysis (SDA). Third and fourth, guanine cytosine (GC) content and the Codon Adaptation Index (CAI) of hospital clade–specific genes were determined.

Overall, 94 of the partially sequenced inserts that were hospital clade–specific were identical or similar to E. faecium DO genes located on 23 to 27 different contigs. Uncertainty in the number of contigs was explained by the presence of multiple copies of the same genes on different contigs, which are predominantly transposases. Most hospital clade–specific genes were dispersed over the different contigs (mostly one gene per contig), but three contigs evidently represented hotspots for hospital–clade specific genes. Twenty-five genes with similarity to the hospital clade–specific ORFs were located on E. faecium DO contig 658, 16 genes were located on contig 656, and nine on contig 653. Clustering of hospital clade–specific accessory genes on the genome may indicate that these gene clusters have been acquired through horizontal gene transfer and recombination.

**Figure 3:**
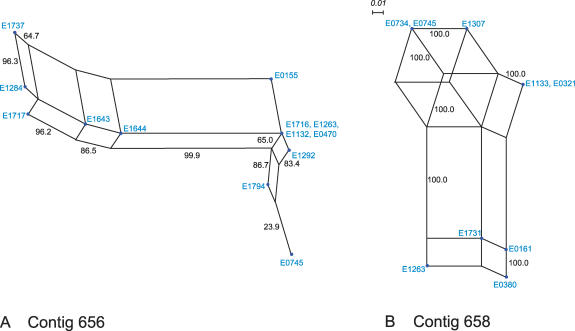
SDA of Hospital Clade–Specific Genes on Contigs 656 and 658 among E. faecium Strains SDA of hospital clade–specific genes on contig 656 of 13 hospital clade strains (A) and hospital clade–specific genes on contig 658 of ten hospital clade strains (B). The nodes represent strains (E numbers correspond to Table 1) and are depicted as blue circles. The scale bar represents Hamming distance. Numbers at the edges represent the percent bootstrap support of the split obtained after 1,000 replicates. Paralellograms indicate recombinatory events. Fit in both graphs is 100%.

**Figure 4:**
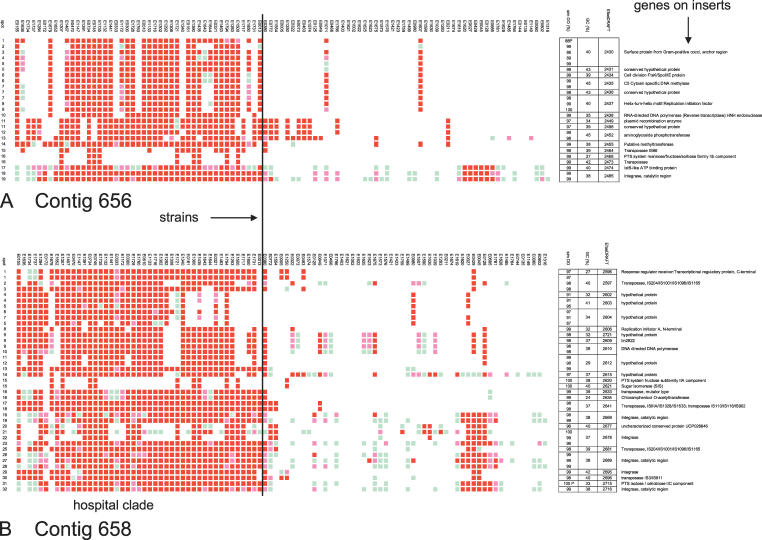
Presence and Absence of Hospital Clade–Specific Genes on Contigs 656 and 658 among *E. faecium* Strains Presence and absence of hospital clade–specific genes on contig 656 (A) and 658 (B) among E. faecium strains. Strains are given on the *x*-axis (E numbers correspond to Table 1). Hospital clade strains are shown on the left panel and are separated from non–hospital clade strains by a vertical line. Hospital clade–specific genes with similarity to E. faecium DO genes, gene numbers, and GC content are given on the *y*-axis on the right side, in presumed order. Spot number is given on the *y*-axis on the left side; more than one gene can be located in one insert. A highly certain present gene (plus 0.5) is indicated with a red square, an absent or highly diverging gene (minus 0.5) with absence of a square. Slightly deviating genes (equal to or larger than 0, and smaller than 0.5) are indicated with a pink square, deviating genes (larger than minus 0.5, and smaller than 0) with a green square.

Phylogenetic analysis of hospital clade–specific genes within contigs 658 and 656 using SDA revealed networked structures consisting of nine parallelograms in contig 656 (*n*(strains) = 13) and eleven parallelograms in contig 658 (*n*(strains) = 9), with reasonable bootstrap values for contig 656 (99.9, 96.3, 96.2, 86.7, 86.5, 65.0, 64.7, 63.4, and 23.9), high bootstrap values for contig 658 (100), and high fit for both contigs (100) (Figure 3). These findings demonstrate the frequent occurrence of recombination events. Several clade-specific genes showed a different GC content than the 36% to 40% found in the rest of the genome (mean 37.9%; http://genome.ornl.gov/microbial/efae): the GC content of five of 16 genes within contig 656 ranged from 34% to 43%, whereas for contig 658, the GC content for eight of 25 genes ranged from 27% to 42% (Figure 4). These findings suggest foreign origin of genes acquired through lateral gene transfer. Inserts that map on the same contig grouped in different clusters based on hierarchical clustering (unpublished data). This indicates that physically linked genes were differentially present in different E. faecium isolates, which is also highly suggestive of genomic mosaicism and recombination events. This is further illustrated in more detail for contigs 658 and 656 in Figures 3 and 4, respectively.

Besides genomic mosaicism, difference in codon usage can support foreign acquisition of genes. The mean CAI in E. faecium core genes was 0.65 (95% confidence interval: 0.62 and 0.68). Five of these genes were all located on contig 595. CAI values of all genes on contig 595 were calculated in comparison with the calculated mean CAI of the E. faecium core, since the whole contig probably contains only E. faecium DO core genes. CAI values were even significantly higher than the E. faecium core CAI (*p* = 0.001, *t*-test), indicating that the calculated mean CAI based on a small number of core genes might be underestimated (Figure S1 and Table S2). Nevertheless, CAI distribution among the contig 656 hospital clade–associated genes and contig 658 hospital-associated genes was significantly lower than mean CAI core genes (*p* < 0.001, *t*-test). However, local variations in CAI in hospital clade–specific gene clusters were substantial (Figure S1). This is exemplified by the CAI of genes belonging to the previously identified putative PAI (Table S3). In this island, the CAI of the *esp* gene (0.71) was higher than the CAI of the other genes (CAI: 0.52–0.62) (Table S3). In conclusion, CAI differences between genes expressed at high and low level as described for E. coli [36] were less pronounced. The relatively high CAI value of E. faecium core genes suggests that the codon usage of E. faecium is shaped towards optimal codon usage irrespective of cellular demands, but that the translational apparatus of the bacterium handled (part of) recruited DNA less efficiently than core DNA. Deviating CAI values and GC percentages of single genes implicate relatively recent acquisition through lateral gene transfer. In summary, these results in E. faecium DO support recent acquisition and recombination of accessory DNA, as defined in this study.

### Character Evolution: Identification of Genes Specific to the Hospital Clade

**Figure 5:**
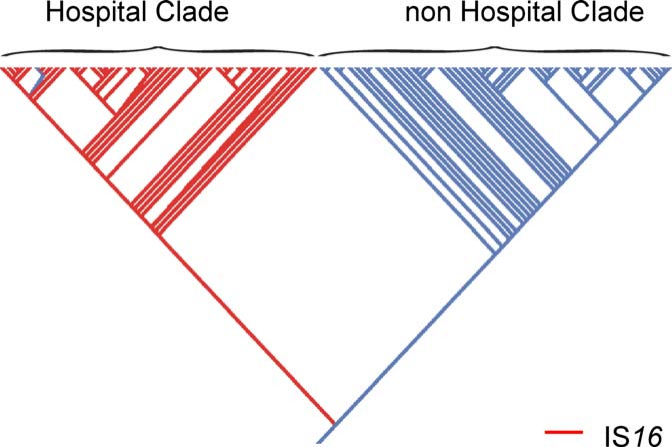
Distribution of *IS*16 among *E. faecium* Strains Maximum likelihood–based gene analysis for determining the distribution of individual IS*16* throughout the phylogenetic tree is shown. Strains in which IS*16* is present are colored red; strains in which IS*16* is absent are colored blue. Strains in the hospital clade all contain IS*16* with one exception. IS*16* is absent in all strains in the non–hospital clade.

Three inserts, all identical to the IS*16* transposase gene (mostly annotated in E. faecium DO as mutator-like transposase), were identified with 98% sensitivity and 100% specificity and validated by Southern blotting as the most clade-predictive locus present in the hospital clade (Figure 5 and Table S4). Multiple copies of this transposase were present in E. faecium DO, though not always likewise annotated. Two complete copies were present in contigs 658 and 646 with 100% and 97% sequence similarity, respectively. Contigs 630, 625, and 546 contained only the right-end side, and contigs 654 and 613 only the left-end side of the gene. IS*16,* part of the IS*256* family, is flanked by nonidentical inverted repeats, with the right inverted repeat resembling a −35 promoter region [37,38]. Sequences of 14 high-ranking hospital clade predictive inserts (≥94% predicted presence and ≤5% presence in the hospital and non–hospital clade ancestral strain, respectively) revealed ≥98% similarity with genes encoding a transposase belonging to the IS*30* family (*tra8* gene) (annotation E. faecium DO: integrase with catalytic region) (*n*(inserts) = 7); an extracellular solute-binding protein, a glycosyl hydrolase, and a conserved hypothetical protein (which are all located on contig 638); chloramphenicol O-acetyltransferase; ROK (Repressor, ORF, Kinase); a phage terminase; and a phage portal protein (Table S4).

Apart from the hospital clade–unique inserts, IS elements were also prominent among hospital clade–enriched inserts. Approximately 30% of all sequenced inserts were similar to genes encoding five additional different types of transposases/IS elements (transposase IS*IIIA*/IS*1328*/IS*1533*; transposase IS*110*/IS*116*/IS*902,* transposase IS*3*/IS*911,* transposase IS*256,* transposase IS*204*/IS*1001*/IS*1096*/IS*1165,* and transposase IS*66*).

Character tracing predicted that the hospital clade acquired certain genes, like the putative PAI, only after initial branching. Absence of the variant *esp* gene in the ancestor of the hospital clade was 98% likely. Other genes acquired after development of the hospital clade include plasmid-derived genes, membrane proteins (*n* = 6), genes involved with carbohydrate transport and metabolism (*n* = 7), transcription-related genes (*n* = 6), defense mechanism genes, aminoglycoside resistance cassettes, and several solitary genes (*n* = 5) not belonging to the same COG.

### Hospital Clade Genome Rearrangements

The observation that IS elements were most specific and abundant for the hospital clade suggests that the acquisition of IS elements increased genome plasticity and the propensity of acquiring further adaptive mechanisms, thus facilitating adaptation to the hospital environment. In general, the genetic variability of isolates that are evolutionary-linked, e.g., the hospital clade isolates, is expected to be less than the genetic variability of isolates that belong to different evolutionary lineages, like all the different non–hospital clade isolates. This difference, however, can be mitigated if specific mechanisms, like the enrichment of IS element, enhance the genetic variability of hospital clade isolates. To compare genetic variation between strains within the hospital clade to variation between all other strains, the genetic similarity among isolates was determined by pulsed-field gel electrophoresis (PFGE), the outcome of which is affected by genome rearrangements, and MLST, which is not influenced by genome rearrangements. As expected, the average genetic similarities among 21 evolutionary-linked hospital clade isolates, based on MLST, was higher (60%) than that among 23 non–hospital clade isolates (26.7%) (Tables S5 and S6). However, the PFGE-based average genetic similarity among the hospital clade strains was 53.6 % ± 13.2 standard deviation (SD), comparable to the average genetic similarity among the non–hospital clade strains (54.9 % ± 12.8 SD; not significant, *t*-test) (Table S7). The resulting recombination/diversity indices of 1.12 ± 0.72 SD for the hospital clade and 0.51 ± 0.50 SD for the non–hospital clade strains supported frequent genome rearrangements in the hospital clade (*p* < 0.001; *t*-test).

## Discussion

Using a mixed whole-genome microarray, we have identified with three different phylogenetic algorithms—Bayesian-based phylogenic analysis, maximum parsimony analysis, and hierarchical clustering—a globally dispersed E. faecium clade among an epidemiologically well-characterized strain collection. This clade is highly specific for nosocomial outbreaks and infections, and 146 clade-specific genes were identified, which were located scattered over 23 to 27 different contigs. Three contigs appeared to be hotspots of these clade-specific genes and were characterized by extensive genomic mosaicim. CAI values and GC content of hospital clade–specific genes on these contigs were slightly different from the rest of the genome. This may indicate rapid adoption of these genes to the translational apparatus of E. faecium or acquisition from closely related species. Among hospital clade–specific genes, IS elements were identified as the most predictive loci for this clade. Besides lateral gene transfer, these IS elements might have facilitated extensive genome rearrangements in the hospital clade as shown by PFGE results, a technique used previously to demonstrate heterogeneity in location of transposon-mediated transconjugation in enterococci [39]. These genomic events could have contributed to the transition of an avirulent commensal to a nosocomial pathogenic E. faecium subspecies.

A random shotgun library of nine E. faecium genomes, selected upon different MLST profiles, was used to investigate population dynamics and genome content of 97 E. faecium isolates. In the absence of a finalized annotation of an E. faecium genome, mixed whole-genome microarray technology offers the optimal tool for comparative genomics. Nevertheless, data interpretation is a potential limitation of microarrays generated upon cloned random fragments rather than gene-specific primers, since multiple gene fragments may be present per insert [40]. This technical restriction stresses the need for validation. Since confirmatory hybridizations matched array data, we consider our microarray suitable for genomic comparisons of isolates.

MLST of E. faecium previously identified host specificity and a globally dispersed subpopulation named CC17, which was associated with hospital outbreaks and infections, and which had apparently replaced the more heterogeneous enterococcal bacterial population within hospitals [22,41,42]. Intriguingly, our microarray phylogenetic analyses, based on genome-wide presence and absence of genes, was comparable to our findings obtained by MLST. Two outbreak-related strains, which were not considered part of CC17 by MLST, clearly belonged to the hospital clade in CGH. Although these strains were evolutionary unrelated to CC17 based on allelic profiles of housekeeping genes, they probably acquired hospital clade–specific genes by horizontal gene transfer. Other E. faecium MLST clonal complexes were not identified by CGH, probably reflecting high recombination frequencies. Congruence between array distance trees and MLST trees has also been described for S. aureus and *Neisseria meningitidis* [43,44].

IS elements contributed prominently to the hospital clade–specific genes. In general, mobile genetic elements, such as ISs, transposons, phages, and genomic islands, are common components of microbial genomes and are driving forces for novel genotypic and phenotypic variants. Transposition of IS elements may disrupt genes, but may also activate downstream genes [37] and fine tune gene expression through transposition mediated genome inversions. Some IS elements contain a −35 promoter-like sequence in their terminal, which may result in formation of a functional promoter [37,38]. Mobile elements are often flanked by IS elements which facilitate recombination and mobilization. The most prominent marker indicative for the hospital clade was IS*16,* which is present in multiple copies on different contigs of the E. faecium DO genome. In the unfinished annotation of E. faecium DO, IS*16* is designated as a mutator type transposase, sharing high similarity with similar tranposases in other bacterial species and in maize [45]. This IS element seems to possess all transposing capacities: IS*16* (*i*) was already identified in E. faecalis as flanking part of Tn*1547* [46], (*ii*) inserted in the *vanY* gene of Tn*1546,* resulting in a VanD-like phenotype in a *vanA* genotype vancomycin-resistant E. faecium [47], and (*iii*) contains a −35 promoter-like sequence. In addition, IS*16* was found in multiple copies in several enterococcal strains [48] and in pRUM, an E. faecium plasmid containing a postsegregational killing system [49]. Our findings demonstrate that IS*16* can be used as a specific marker of the hospital clade genotype.

Enrichment of particular IS elements in the genome of bacterial (sub)species has been documented previously. In *S. epidermidis,* IS*256* is present in multiple copies in clinical strains, where it might induce genome flexibility of multiresistant, biofilm-forming isolates [33]. *Shigella* species are enriched with 300 to 700 copies of IS elements [50], and Bordetella pertussis is significantly enriched in IS elements compared with *Bordetella bronchiseptica,* from which B. pertussis is thought to have been evolved [51,52]. The observation that IS elements are among the most specific and abundant hospital clade–specific inserts suggests that acquisition of these elements has contributed to the ecological success of this clade in the hospital environment, through enhanced genome plasticity*.* A higher diversity index for the hospital clade compared with that of the non–hospital clade demonstrates higher levels of genetic variability, which could have resulted from enrichment with IS elements. IS elements may not only affect gene expression and enhance genome plasticity, but may also increase the propensity of acquiring further adaptive mechanisms. All of these IS element–induced events may have been pivotal for E. faecium to adapt and survive in highly competitive niches, such as the hospital environment.

The structure and genetic makeup of the E. faecium pangenome shows many similarities with the sequenced E. faecalis V583 [53]. In concordance with the more than 25% mobile elements in V583, 50 (putative) IS elements were contained among the 146 identified E. faecium hospital clade–specific genes. One of the most prominent V583 IS elements, IS*256,* was also found among these. V583 plasmids seem to be complex mosaic structures compared with similar plasmids in GenBank [53]. The presence of multiple plasmid remnants and the three resident plasmids in V583 was hypothesized as being important for genome plasticity [53]. Our data for contigs 656 and 658 provide more evidence for this concept in E. faecium.

Highly prevalent hospital clade–specific genes represent antibiotic resistance genes, present in 80%–83% of the hospital clade isolates, and genes involved in carbohydrate metabolism, present in 89%–93% of hospital clade isolates, in addition to IS elements. Interestingly, we found the *aadE-sat4-aphA-3* aminoglycoside streptothricine resistance gene cluster highly associated with the hospital clade (present in 84% of hospital clade isolates) and not linked to a specific IS element. In a previous study, this gene cluster had been identified as part of a Tn*5405*-like structure in 70.1% of isolates [54]. The Tn*5405*-like structure is present in E. faecium DO, with IS*1182* annotated as EfaeDRAFT_2457 cassette chromosome recombinase B1 on contig 656, and ORFs X and Y (EfaeDRAFT_2456 and 2455) and the *aadE-sat4-aphA-3* cluster (EfaeDRAFT_2452, 2453 and 2454) located directly upstream. In contrast to our data, this gene cluster was found in German multiresistant animal and sewage isolates, as well as in human hospitalized patients and outpatients [55]. Most hospital clade–specific hypothetical proteins and other genes, including the putative PAI genes, were identified in only a smaller subset, reflecting acquisition after initial branching of the hospital clade. Previously, the PAI has also been identified only in a fraction (approximately 60%) of hospital outbreak and infection strains [22]. Frequent recombination resulted in genomic mosaicism, as exemplified by contigs 656 and 658. Variation in the combination of accessory genes might supply the organism with a varying armamentarium to colonize or infect the host and escape the immune system.

Our study is unique in that it identifies a single phylogenetic hospital clade of strains with epidemic potential in hospitals *and* with more than 100 genes specific to this clade. Among the 97 strains included in this study, a core genome of 65% of the inserts was identified, while 13% of spots were highly associated with the hospital clade. Genetic subpopulations strongly associated with virulent or epidemic potential have not been identified for many other species. Recently, a nonlivestock-associated clade that contained all infectious Campylobacter jejuni isolates and associated genetic factors was identified using an approach similar to ours [56]. In addition, Howard et al. confirmed by comparative phylogenomics three distinct clusters of Yersinia enterocolitica composed of a nonpathogenic clade, a low pathogenic clade, and a high pathogenic clade [57]. In contrast, population genetics of S. aureus repeatedly failed to identify virulence factors associated with enhanced virulence or a subpopulation adapted to the hospital, though overrepresentation of invasive isolates in certain (sub)clusters was identified [28,30,58]. In another study, factors associated with S. epidermidis invasiveness were identified, but a phylogenetic analysis failed to distinguish invasive isolates from controls [33]. Nevertheless, evolution of a pathogenic species from a less pathogenic species has been documented before (i.e., *Shigella* species from E. coli [59], and Bacillus anthracis from Bacillus cereus [60]). A quantum leap of evolution in these bacteria occurs when new genes are acquired en bloc via horizontal gene transfer by plasmids and bacteriophages [61]. Acquisition of these genes enables the pathogen to colonize a new niche, and new selective constraints lead to progressive adaptation of the organism by pathoadaptive mutations [62]. When aligning the enterococcal plasmid pRUM to E. faecium DO, the plasmid was highly similar to parts of contig 658, including the toxin–anititoxin system. This may reflect integration of a plasmid in the E. faecium genome. An important element of pathoadaptive evolution is selection of “black holes”: inactivation to pseudogenes or loss of genes, which leads to genome decay [62]. The inactivated and lost genes are often antivirulence genes [62]. This DNA may still be present in the nonpathogenic ancestor. Our results didn't show direct evidence for complete loss of many genes specific for hospital clade strains; however, identification of pseudogenes with this approach is not possible. The fact that hospital clade strains are only rarely found outside the hospital indicates reduced fitness of these strains in the ancestral niche, which possibly reflects antagonistic pleiotropy in which hospital specialization is detrimental in other niches [63]. From these evolutionary insights, one might conclude that worldwide emergence of the E. faecium hospital clade represents, in fact, evolution of a novel hospital-adapted subspecies from a nonpathogenic (commensal) E. faecium ancestor, which succeeded in competing with E. faecalis as causative agent of hospital infections.

## Materials and Methods

### Bacterial strains and nucleic acid extraction.

The 97 bacterial isolates used in this study originated from different documented epidemiological niches: 18 mostly monoclonal, hospital epidemics; 35 clinical sites representing invasive human infections, including E. faecium DO (E1794 in Table 1); 15 hospital surveys, representing asymptomatic carriage of hospitalized patients; 11 community surveys, representing asymptomatic carriage in healthy subjects; three environmental isolates; and 15 animals in 21 countries on six continents (Table 1) [20,41,64–72]. These strains were a subset of a large, genotypically well-characterized collection, which represented the global E. faecium population. The subset was selected based on differences in geographic locations, hosts, and sequence types. All strains were cultured on tryptic soy agar sheep blood plates at 37 °C. DNA for labeling (see below) was prepared from cell suspensions by bead-beating and chloroform phenol extraction. DNA for Southern blots was isolated according to the manufacturer's instructions with DNeasy Tissue kit (Qiagen, http://www.qiagen.com). The shotgun library was created from nine strains from different epidemiologic and genetic backgrounds according to MLST analysis (Table 3) [21]. In order to prevent overrepresentation of inserts from high copy plasmids, plasmid DNA was separated from chromosomal DNA according to Willems et al. [73] with the modification that clumping high molecular chromosomal DNA, cured from plasmid DNA, was captured with a glass capillary. Plasmids were prepared from the library strains with the QIAprep Spin Miniprep Kit according to the manufacturer's instructions (Qiagen) and amplified with the TempliPhi Amplification Kit (Amersham, http://www.amersham.com).

**Table 3:**
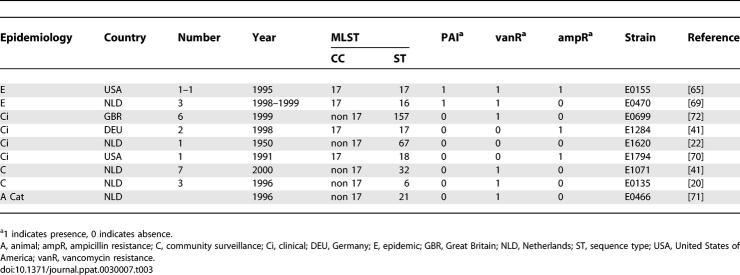
Strains Used in Library

### Microarray fabrication.

Equal amounts of chromosomal DNA from nine genetically diverse E. faecium strains were mixed to create a shotgun library as described by Borucki et al. [74] (Table 3). The same procedure was repeated for the plasmid DNA preparations. Briefly, 10 μg of pooled DNA (equal amounts from each strain) was sonicated (Branson 250/450 Sonifier, 6-mm microtip; http://www.sonifier.com), and fragments of approximately 0.8–1.2 kb for genomic DNA and 1.2–1.7 kb for plasmid DNA were gel isolated, extracted (Qiaquick columns, Qiagen), and end-repaired (DNA Terminator End Repair Kit; Lucigen Corporation, http://www.lucigen.com). End-repaired fragments were ligated to pSMART-HC-Kan (Clone-SMART, Lucigen), and E. coli (ElectroMAX DH10B Cells; Invitrogen, http://www.invitrogen.com) were transformed with this recombinant plasmid. Next, 4,560 genomic and 1,140 plasmid recombinant clones were arrayed in 96-well plates. Clone inserts were amplified by PCR with amino-modified SMART primers. Additionally, fragments from one enterococcal housekeeping gene, 13 virulence genes, and 20 genes involved with antibiotic resistance were PCR–amplified and included in the microarray (Table S7) [18,49,55,75–89]. PCR products were ethanol purified and resuspended in 1 × SSC (1 × SSC is 0.15 M NaCl plus 0.015 M sodium citrate). All genomic, plasmid, and additional gene PCR products were printed by using ESI three-axis DB-3 robot (Versarray ChipWriter Pro; Biorad, http://www.bio-rad.com) at a controlled humidity of 55% on CSS silylated slides (European Biotech Network, http://www.euro-bio-net.com). Slides were printed in two batches, after which they were blocked following the manufacturer's instructions. Genomic coverage of the library on the nucleotide level was calculated using Formula 1 [35]:

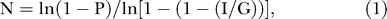
in which N = number of clones, P = probability of coverage, I = insert size and G = genome size.

This formula, however, is based on the assumption that that every single nucleotide should be present in the library. In the present approach, there is no need for a complete ORF to be present: border sequences with a minimal size of 100 nucleotides should be sufficient to obtain positive hybridization signals (Formula 2) [34]:

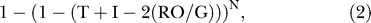
in which T = transcript length and RO = required overlap.

In this study, both algorithms are used to estimate genomic coverage.

### Labeling, hybridization, and data acquisition.

Total DNA (0.5 μg) was labeled with fluorescent dyes by random priming with the Bioprime labeling system (Invitrogen). To normalize the two channels for label incorporation, DNA concentration differences, and variation in slide scanning, equal amounts of the library strains were mixed as the reference pool and labeled with Cy3 dUTP. Tester strains were labeled with Cy5 dUTP. Ten tester strains were hybridized in duplo for control of reproducibility. For each hybridization, Cy5 and Cy3 probes were combined with yeast tRNA, speed vacuum dried, resuspended in 40 μl Easy hyb buffer (Roche Diagnostics Netherlands B.V., http://www.roche-diagnostics.nl), and denatured for 2 min at 100 °C.

Silylated slides were prehybridized in prehybridization solution (1% BSA, 5 × SSC and 0.1% sodium dodecyl sulfate, filtered) at 42 °C during 45 min while rotating, washed twice with filtered milli Q water, dried with N_2_ flow, and prewarmed at 42 °C. Easy hyb solution was pipetted on the microarray print of the slide, covered with a hybrislib, and placed in hybridization chambers (Corning Life Sciences B.V., http://www.corning.com/lifesciences). Hybridizations were performed overnight at 42 °C in a waterbath. Microarrays were then washed sequentially in (*i*) 1 × SSC/0.2% sodium dodecyl sulfate for 10 s at 37 °C, (*ii*) 0.5 × SSC for 10 s at 37 °C, and (*iii*) twice in 0.2 × SSC for 10 min at room temperature, and dried with N_2_-flow. A Scanarray Express 680013 Microarray Analysis System (PerkinElmer Life and Analytical Sciences, http://las.perkinelmer.com) was used for scanning slides.

Microarray images were quantified with Imagene software version 4.2 (Biodiscovery, http://www.biodiscovery.com). Inferior spots (empty spots, exceeding SD of pixels, less than two times background in Cy3 channels), were excluded from normalization and data analysis.

### Data processing, comparative phylogenomics, and identification of predictive genes.

To correct for differences in labeling, hybridization conditions, slide quality, and scanning circumstances, each slide was normalized independently. At first, ratios of Cy5 minus background to Cy3 minus background were calculated and log2-transformed. Filtering was applied to exclude spots with flags; for estimating the correction factor in normalization, only spots were included with Cy3 values larger than two times background. Mean log2 ratios were calculated and applied to each independent ratio. Next, the data were transformed using GACK (http://falkow.stanford.edu/whatwedo/software/software.html) to assign a region of considerate absence and presence, corresponding with, respectively, minus 0.50 and plus 0.50, and an interval with indefinite presence/absence, or divergence, to interpret the data. For Bayesian modeling, maximum parsimony analysis, SDA, and character evolution modeling data was transformed to binary output.

Using a Nexus format matrix, the relationship of strains based on the presence and absence of hybridizing signal on spotted inserts was determined with Bayesian-based algorithms implemented through MR BAYES 3.0 software [90], as explained by Champion et al. [56]. With samples and saves from every 40th tree, 1,100,000 generations of four incrementally heated Markov chain Monte Carlos were performed on the DNA–DNA microarray data by using the default annealing temperature of 0.5, a burn-in of 100,000 Markov chain Monte Carlos generations, and an 8-category distribution. Ninety five percent majority rule consensus trees and clade credibility values were obtained by using TreeView (http://taxonomy.zoology.gla.ac.uk/rod/treeview.html). In addition to the Bayesian-based approach, phylogeny was studied with hierarchical clustering and maximum parsimony analysis. [24]. Bootstrapped (1,000 iterations) complete linkage transversal hierarchical clustering with Euclidian distance was performed and visualized with TIGR MeV version 3.1 software (http://www.tm4.org/mev.html). One thousand times bootstrapped maximum parsimony analysis was performed with PAUP* 4.0 software (Sinauer Associates, http://paup.csit.fsu.edu).

In order to select a maximal variety of differential genes and gain insight into the degree of redundancy, hierarchical clustering (for details see above) was used to generate a dendrogram of genes based on their patterns of absence and presence across the strains. Detection of clusters of acquired genes is based on the assumption that co-inherited genes can be found co-located on the bacterial genome. Subsequently, a subset of inserts was considered highly specific for a clade according to the following two criteria: First, insert specificity for the clade was higher than 80%, estimated with the χ^2^ test and followed by FDR correction [91]. Second, the insert clustered with a Euclidian distance of 1.1 or less for the genomic library and 0.8 or less for the plasmid library was selected for sequencing (see below). To gain insight into the core genome; 35 randomly chosen inserts that gave a positive hybridization signal in all strains were sequenced.

SDA based on the presence and absence of ORFs, with identity to genes belonging to contigs 658 and 656 in a selection of the most genetically diverse strains, was used to test for parallel changes in the gene order on these E. faecium DO contigs. The bootstrapping procedure for SDA was used as implemented in the SplitsTree program version 4.0 using Hamming correction (http://www.splitstree.org). Recombination, hybridization, gene conversion, and gene transfer all lead to histories that are not adequately modeled by a single tree. They effectively cause lineages to coalesce forward in time, resulting in trees that have reticulations or a network structure rather than the simple branching structure seen with most phylogenies. Split decomposition does not force tree topologies to be strictly bifurcating or multibranching but permits network relationships. A split decomposition graph will look less tree-like and more net-like as the influence of recombination becomes more important in the history of a set of taxa. Since splits graphs were sufficiently complex and the distances among strains sufficiently great, data sets had to be simplified by removing strains representing the longest branches to allow visualization of central networks and improve the fit parameter, which is similar to the method described in [92].

The character evolution maximum likelihood–based model of Mesquite 1.06 software (http://www.mesquiteproject.org) was used to identify clade-predictive genes on binary data in nexus format. Tracing shows the most likely hypothesis of ancestral states, and indicates how presence and absence or divergence of certain genes in an ancestral strain has led to the formation of a new clade.

### Sequencing and analysis of differential inserts and validation of microarray results.

Inserts selected for sequencing (see above) were PCR-amplified and sequenced single-stranded from one direction in combination with the BigDye Terminator reaction kit by using an ABI PRISM 3700 DNA analyzer (Applied Biosystems, http://www.appliedbiosystems.com). All sequences were blasted in GenBank. COGs for E. faecium genes and proteins were assigned according to the Oak Ridge National Laboratory Web site (http://maple.lsd.ornl.gov/cgi-bin/JGI_microbial/display_page.cgi?page=cog&org=efae&chr=08jun04). COGs for other species' genes and proteins were assigned according to GenBank.

Microarray hybridization results from ten spots, including six different ORFs (IS*16, esp, vanA,* transposase IS*111A*/IS*1328*/IS*1533*; transposase IS*110*/IS*116*/IS*902, glycosyl hydrolase family 88,* and *extracellular solute-binding protein*), were validated by Southern hybridization. For this purpose, chromosomal DNA preparations were digested with EcoRI, separated by agarose gel electrophoresis (0.8% agarose gels), transferred onto a Hybond N^+^ nylon membrane (Nycomed Amersham plc, http://www.amersham.com), and subsequently hybridized to six ECl-labeled PCR products specific for the six ORFs according to the manufacturer's protocol (Amersham). Primers, if not already designed for the additional spots on the array (Table S7; see “Microarray fabrication” section of Materials and Methods), were listed in Table S8.

### Determining the codon adaptation index and GC content.

Codon usage patterns may vary considerably among genes. It is generally assumed that codons that are best recognized by the most abundant tRNA are those that are translated optimally (most accurate), and are often linked to genes expressed at high levels. The CAI measures codon bias and indicates the relative adaptiveness of the codon usage of a particular gene towards the codon usage of highly expressed genes. A gene that consists only of the most frequently used codons of a reference set of highly expressed genes has the maximal possible CAI value of 1.0 and is thought to be highly expressed. In general, highly expressed genes have high CAI values. The CAI adaptation index was calculated as described previously [36]. Briefly, a CAI calculator (http://www.evolvingcode.net/codon/cai/cai.php) was used to determine the relative synonymous codon usage (RSCU)—that is, the observed frequency of a particular codon divided by its expected frequency under the assumption of equal usage of the synonymous codons for an amino acid [36]—of analyzed genes. CAI is defined as the geometric mean of the RSCU values corresponding to each of the codons used in that gene, divided by the maximum possible CAI for a gene of the same amino acid composition [36]. A subset of E. faecium DO core genes representing genes encoding elongation factors and ribosomal proteins was supposed to be highly expressed in E. faecium and was used as a reference for RSCU (Table S9). CAI values were then calculated using the CAI calculator (http://www.evolvingcode.net/codon/cai/cai.php). The DRAFT annotation of the E. faecium DO genes is from the Joint Genome Institute E. faecium Web site (http://genome.jgi-psf.org/draft_microbes/entfa/entfa.home.html). The mean CAI value with SD was calculated from the sequenced core genome genes (Table S1). Furthermore, the known putative E. faecium PAI genes were included. The observed codon frequencies in the E. faecium genome were compared with the expected codon frequencies calculated from the GC content at the first, second, and third codon positions under the assumption of the same amino acid composition. The significance of the differences was evaluated by *t*-test statistics.

### PFGE and diversity index.

PFGE analysis was performed as described previously [6]. Distance matrices of banding patterns between 339.5 kb and 97 kb were calculated with Bionumerics software (version 3.5; Applied Maths, http://www.applied-maths.com) by the Ward method for a subset of hospital clade strains (*n* = 22) and a subset of non–hospital clade strains (*n* = 24) with different sequence types and epidemiological origins. Distance matrices from previously identified MLST allelic profiles were calculated with the categorical coefficient (Bionumerics software). In order to compare the level of similarity among isolates belonging to the hospital clade with non–hospital clade isolates based on PFGE and MLST, a so-called diversity index was calculated. For this, the average of similarities between all possible pairs of hospital clade and non–hospital clade isolates based on MLST was calculated and divided by the average of similarities between all possible pairs of hospital clade and non–hospital clade isolates based on PFGE. When the level of genetic diversity (is 1− similarity) based on MLST and PFGE is identical, the diversity index equals 1. A diversity index greater than one indicates that the average pairwise similarity based on PFGE is higher than that based on MLST, and suggests enhanced genomic rearrangements.

## Supporting Information

Dataset S1 Flag Filtered, Normalized Log-Transformed E. faecium Mixed Genome Array Data (4.4 MB TXT)

Figure S1 CAI and GC Content of Core Genes on Contig 595 and Hospital Clade–Specific Genes on Contigs 656 and 658 Black lines represent the CAI of hospital clade–specific genes. Dark blue lines indicate hospital clade–specific IS elements with no corresponding CAI value. The mean CAI of the core genes is represented by the red straight lines; the red dotted lines indicate the 95% confidence interval.
(27 KB PDF)

Table S1 E. faecium Core Genome Sequenced Genes (24 KB XLS)

Table S2 CAI Values of E. faecium DO Sequenced Core Inserts (15 KB XLS)

Table S3 CAI Values of Putative PAI Genes (13 KB XLS)

Table S4 Ranking of Inserts Based on Predominance of Hospital Clade Ancestral State, Identified from Character Evolution Modeling (299 KB XLS)

Table S5 Distance Matrices of Banding Pattern Similarity of Hospital Clade and Non–Hospital Clade Strains Obtained from PFGE (20 KB XLS)

Table S6 Distance Matrices of Similarity of MLST Allelic Profiles of Hospital Clade and Non–Hospital Clade Strains (20 KB XLS)

Table S7 Primers Used for PCR-Amplified Antimicrobial Resistance, Virulence, and Housekeeping Genes a, antimicrobial resistance; h, housekeeping genes; v, virulence.
(29 KB XLS)

Table S8 Primers Used for Validation Array (13 KB XLS)

Table S9 E. faecium DO Elongation Factors and Ribosomal Proteins Used to Calculate Reference RSCU (15 KB XLS)

### Accession Numbers

The GenBank (http://www.ncbi.nlm.nih.gov/Genbank/index.html) accession numbers for the genes and gene products discussed in this paper are E. faecium isolate E300 pathogenicity island (AY322150), pEFNP1 (AB038522), and pKQ10 (U01917).

## Abbreviations

CAI: Codon Adaptation Index
CC: clonal complex
CGH: comparative genomic hybridization
COG: cluster of orthologous genes
*esp*: *enterococcal surface protein*
FDR: false discovery rate
g: genomic
GC: guanine cytosine
IS: insertion sequence
MLST: multi locus sequence typing
ORF: open reading frame
p: plasmid
PAI: pathogenicity island
PFGE: pulsed-field gel electrophoresis
PP: Bayesian posterior probabilities
RSCU: relative synonymous codon usage
SD: standard deviation
SDA: split decomposition analysis
VRE: vancomycin-resistant enterococcus
