# Supplementary figures and images for: Insertion Sequence–Driven Diversification Creates a Globally Dispersed Emerging Multiresistant Subspecies of E. faecium
Source: PLoS Pathog. 2007 Jan 26;3(1):e7. doi: 10.1371/journal.ppat.0030007 (PMC1781477; doi:10.1371/journal.ppat.0030007)

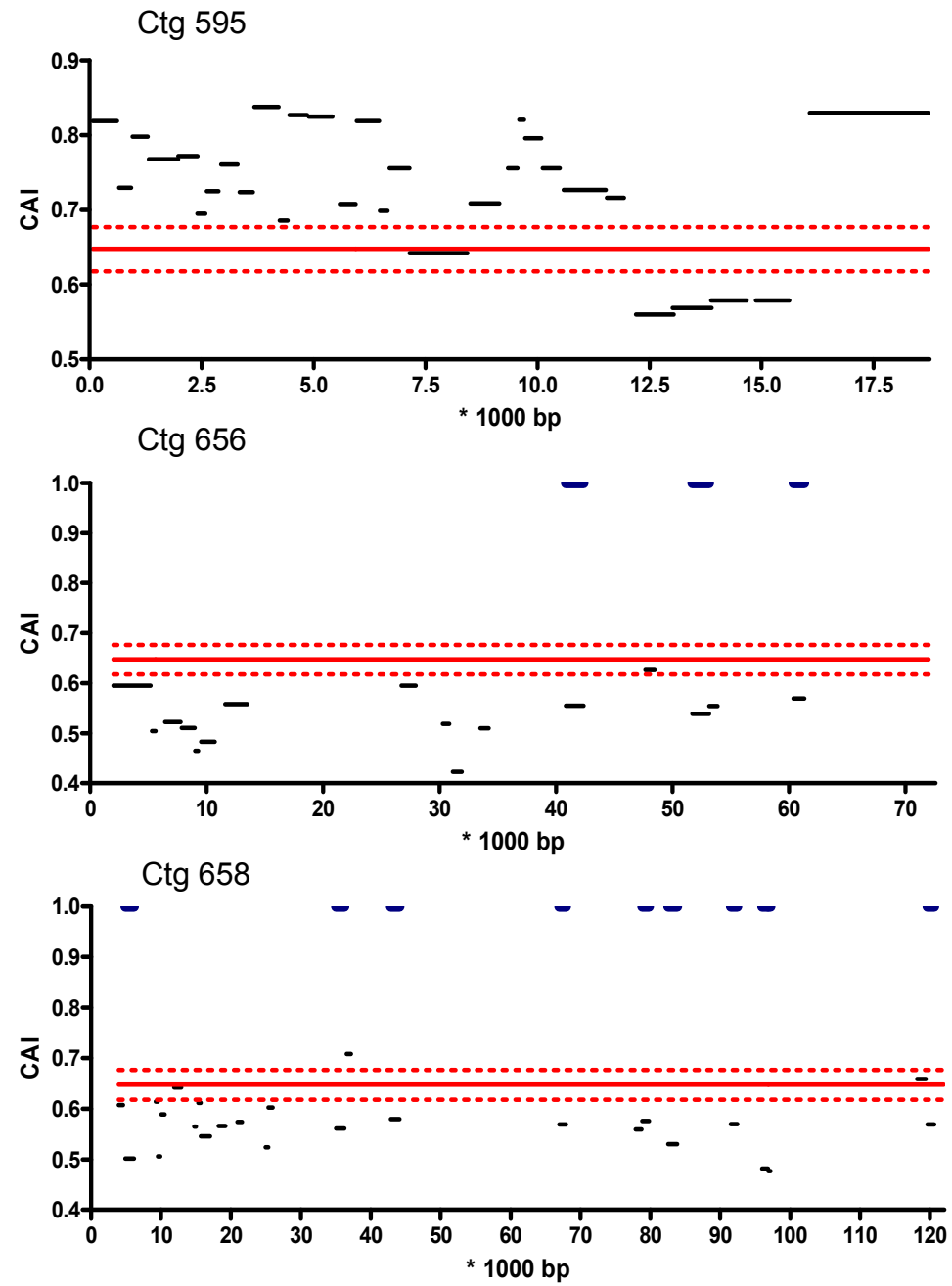

Figure S1

Supplement: Figure S1 — Black lines represent the CAI of hospital clade–specific genes. Dark blue lines indicate hospital clade–specific IS elements with no corresponding CAI value. The mean CAI of the core genes is represented by the red straight lines; the red dotted lines indicate the 95% confidence interval. (27 KB PDF) [file ppat.0030007.sg001.pdf]
